# The effect of age on the association between daily gait speed and abdominal obesity in Japanese adults

**DOI:** 10.1038/s41598-021-98679-1

**Published:** 2021-10-07

**Authors:** Keita Kinoshita, Naoki Ozato, Tohru Yamaguchi, Motoki Sudo, Yukari Yamashiro, Kenta Mori, Mika Kumagai, Kaori Sawada, Yoshihisa Katsuragi, Seiya Imoto, Kazushige Ihara, Shigeyuki Nakaji

**Affiliations:** 1 Department of Active Life Promotion Sciences, Graduate School of Medicine, Hirosaki University, Aomori, Japan; 2 Health & Wellness Products Research Laboratories, Kao Corporation, Tokyo, Japan; 3 Personal Health Care Products Research Laboratories, Kao Corporation, Tokyo, Japan; 4 Human Genome Center, Institute of Medical Science, University of Tokyo, Tokyo, Japan; 5 Department of Social Medicine, Graduate School of Medicine, Hirosaki University, Aomori, Japan

**Keywords:** Health care, Public health, Weight management

## Abstract

The aim of this work was to investigate the effect of age on the association between daily gait speed (DGS) and abdominal obesity defined by visceral fat area (VFA). A cross-sectional study was performed using data from an annual community-based health check-up. A total of 699 participants aged 20–88 years were enrolled in this analysis. DGS was assessed using tri-axial accelerometers worn for ≥ 7 days with at least 10 measuring hours each day. VFA was measured using a visceral fat meter. Since DGS differed significantly with age, the participants were divided into two groups: younger adults (YA), aged 20–49 years, and older adults (OA), aged 50–88 years. The association between DGS and VFA differed significantly with age (r = 0.099 for YA and r =  − 0.080 for OA; test for difference between correlation coefficients, *P* = 0.023). In OA, the adjusted odds ratio of abdominal obesity (VFA ≥ 100 cm^2^) was 0.40 (95% confidence interval 0.18, 0.88, *P* = 0.022) for the highest DGS quartile (DGS ≥ 1.37 m/s) compared to that for the lowest quartile (DGS < 1.11 m/s), whereas no significant association was found in YA. These data could aid in raising awareness of the self-management of obesity via DGS monitoring, especially in OA.

## Introduction

The incidence of obesity has been increasing over the past decades worldwide^1^. Obesity is thought to be a cause of multiple health problems^2^. Although body mass index (BMI) is frequently used in clinical settings to assess obesity status (general obesity), recent studies have shown that abdominal obesity, defined by visceral fat area (VFA), has stronger associations with hypertension, type 2 diabetes, dyslipidaemia, and cardiovascular disease than BMI^2–4^. Therefore, reducing the VFA might be more effective at decreasing the risk of these diseases than reducing the BMI. Waist circumference is a simple and convenient indicator that reflects VFA, and it is used as one of the components of metabolic syndrome by several organisations globally^5^.

Obesity is caused by an imbalance between energy intake and energy expenditure. Walking might account for up to 30% of an adult’s daily energy expenditure^6^ and is therefore important in preventing obesity^7,8^. Gait speed, a key indicator of functional abilities in individuals^9,10^, is directly related to energy expenditure during walking^11–13^. Several studies have reported that decreased gait speed is associated with abdominal^14–16^ or general obesity^17,18^ in elderly subjects. However, Moreira et al.^19^ demonstrated no significant association between gait speed and abdominal obesity in middle-aged subjects. Schimpl et al.^20^ reported no significant association between gait speed and general obesity in their subjects aged 17–65 years. Therefore, the association between gait speed and abdominal or general obesity remains to be conclusively determined.

One of the possible explanations for this inconsistency is that the association between energy expenditure and gait speed was confounded by the subjects’ ages. A recent meta-analysis study showed that elderly subjects (mean age ≥ 59 years) expend more metabolic energy than younger subjects (mean age 18–41 years) when walking at comparable speeds^6^, suggesting that individuals need to be classified by age. Another possible explanation is that gait speed was assessed in laboratory settings in previous studies. Takayanagi et al.^21^ reported that the association between in-laboratory gait speed and daily gait speed (DGS) was weak. In terms of energy expenditure when walking during daily life, DGS might be a better indicator of the risk of obesity than in-laboratory gait speed.

In general, older people are defined as individuals aged ≥ 60 or ≥ 65 years; however, there is insufficient evidence for the association between DGS and age or obesity. The aim of this study was to investigate the effect of age on the association between DGS and abdominal obesity, as well as general obesity, in Japanese adults.

## Methods

### Participants

The Iwaki Health Promotion Project was launched in 2005 as an annual health check-up program. Participants were adult men and women living in the Iwaki region of Hirosaki City in Aomori Prefecture, Japan^22–24^. This study was designed as a population-based cross-sectional study, using data obtained from a 2018 health check-up. In 2018, 1056 individuals participated in the health check-up from May 27 to June 5. Of these, 120 did not complete the clinical assessments, dietary data, or accelerometer data, and therefore, were excluded from the analyses. In addition, we excluded 237 participants who did not meet the criteria of accelerometer data. The criterion for the analysis was wearing the accelerometer on the waist for a total duration of ≥ 7 days, for ≥ 10 h/day, during the first 10 days after beginning to wear the accelerometer^21,25,26^. In total, 699 participants, 264 men, 435 women aged 20–88 years, were included in the analysis.

The study was approved by the Ethics Committee of Hirosaki University School of Medicine and conducted in accordance with the principles of the Declaration of Helsinki (2018-012, 2018-063). Written informed consent was obtained from all the participants prior to the study. This study was registered in the University Hospital Medical Information Network (UMIN-CTR, https://www.umin.ac.jp, UMIN ID: UMIN000036741). This study was conducted in accordance with the Strengthening the Reporting of Observational Studies in Epidemiology (STROBE) guidelines.

### DGS measurement

DGS was continuously measured on a daily basis using a tri-axial accelerometer (HW-100, Kao Corporation, Tokyo, Japan)^21^. The accelerometer provides 40 days of continuous recording at a sampling frequency of 64 Hz^25^. This device detects the step cycle during gait, ranging from 70 to 160 steps/min, via medio-lateral and vertical acceleration. It commences recording tri-axial acceleration during the gait cycle if the measured acceleration of the current cycle and the two preceding cycles are within 10% of one another. Therefore, the accelerometer records tri-axial acceleration during the steady-state periods of gait. DGS was calculated using a model that used composite acceleration during one gait cycle from the tri-axial acceleration measurements. An average DGS was obtained for valid days during a 10-day period for each participant. The accelerometer also measured wearing time and step counts^25^.

The participants were instructed to wear the HW-100 on their waists at all times while they were awake, except during swimming or bathing, and to maintain their usual activities. Additionally, the participants were instructed to start wearing the HW-100 soon after their health check-up was completed and to return it after 10 days.

### Measurement of other parameters

VFA was measured using a bio-impedance type visceral fat meter^24,27^, EW-FA90 (Panasonic Corporation, Osaka, Japan), which is a certified medical device in Japan (No. 22500BZX00522000) that measures VFA in a non-invasive way. The measurements obtained by this device correlate highly with those obtained using computed tomography^27^, the gold standard for VFA measurement. The following clinical characteristics were also measured: height, body weight, BMI, serum glucose, haemoglobin A1c, systolic blood pressure, diastolic blood pressure, low-density lipoprotein cholesterol (LDL cholesterol), high-density lipoprotein cholesterol (HDL cholesterol), and serum triglyceride levels. Blood samples were collected from the peripheral veins. All laboratory measurements were outsourced to LSI Medience Co. (Tokyo, Japan) and conducted according to their standard operating procedure. Data on smoking habits and medications were procured through questionnaires prepared for the health check-up. Daily intake of energy and alcohol was calculated using the Brief Diet History Questionnaire^28^.

Abdominal obesity was defined as VFA ≥ 100 cm^2^ in the cross-section of umbilical level and general obesity was defined as BMI ≥ 25 kg/m^2^, according to the definition published by the Japan Society for the Study of Obesity^29^. Hypertension was defined as blood pressure ≥ 140/90 mmHg or the use of antihypertensive drugs^30^. Diabetes was defined as fasting serum glucose ≥ 126 mg/dL, HbA1c ≥ 6.5%, or the use of antidiabetic drugs^31^ Dyslipidaemia was defined as LDL cholesterol ≥ 140 mg/dL, HDL cholesterol < 40 mg/dL, triglycerides ≥ 150 mg/dL, or the use of antihyperlipidemic drugs^32^.

### Statistical analysis

Participant characteristics are reported as means ± standard deviations (SDs) or percentages. Mann‒Whitney U tests were used when the number of participant groups was two, whereas for comparisons of more than two participant groups, Cochran‒Armitage trend tests were used. Chi-square tests and two-sample proportion tests were used for categorical variables. For comparisons between groups, Cohen’s d was calculated as a measure of effect size. The overall relationship between age and DGS was evaluated using several regression models (e.g. polynomial and spline) to calculate the cut-off age for DGS decline. Smoothing spline, which is commonly used for building explanatory models in clinical research^33^, was chosen because of goodness-of-fit-based on Akaike information criterion. The spline curve was fitted with the optimal value of the smoothing parameter, determined by a generalised cross-validation method^34^. To evaluate the association between DGS, age, VFA, and BMI, we used Spearman’s correlation coefficient and partial correlation coefficient and assessed the differences between the correlation coefficients. Gait speed is known to be positively associated with the number of daily steps^35^; therefore, a partial correlation between gait speed and age was adjusted by daily steps and sex. Multiple logistic regression analysis was performed to investigate the adjusted odds ratio (aOR) and 95% confidence interval (CI) for abdominal and general obesity status, comparing participants in the higher quartiles of DGS to those in the lowest quartile. DGS was also analysed as a continuous variable (per 0.1 m/s). VFA is associated with daily steps^36^, smoking^37^, alcohol intake^38^, and lifestyle-related diseases such as hypertension, diabetes, and dyslipidaemia^2–4^. We performed logistic regression analysis considering these factors. Model 1 was adjusted for sex and age, model 2 was further adjusted for alcohol consumption (g/day), smoking habit (cigarettes/day), and total energy intake (kcal/day), model 3 was additionally adjusted for average steps (/day), and model 4 was adjusted in a manner similar to model 3 along with incorporation of hypertension, diabetes, and dyslipidaemia. Statistical tests were two-tailed, and results with *P* < 0.05 were considered significant. All analyses were performed using SPSS (version 25; SPSS Inc., Chicago, IL, USA) and R (version 3.6.2; R Core Team, Vienna, Austria).

## Results

**Figure 1:**
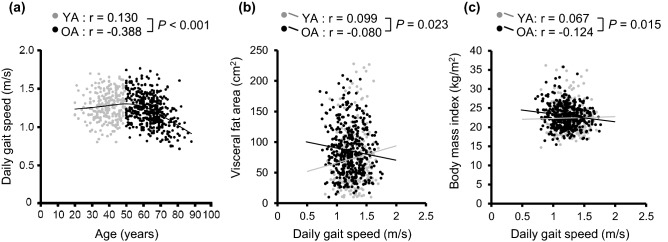
Association between daily gait speed (DGS) and age or visceral fat area (VFA) or body mass index (BMI). (**a**) Scatter plot with smoothing spline for the relationship between age and DGS (n = 699). The vertex was 49.8 years. (**b**,**c**) Scatter plots with linear regression lines for the relationship between DGS and VFA or BMI. Grey circles and lines represent younger adults (YA; age 20–49 years, n = 255) and black circles and lines represent older adults (OA; age 50–88 years, n = 444). The test for the group differences between the correlation coefficients was performed using Spearman’s correlation coefficient.

The relationship between DGS and age of the study participants (n = 699) is presented in Fig. 1a. The relationship was investigated using a smoothing spline, in which the vertex was at the age of 49.8 years. The relationship between age and DGS was significantly different before (younger adults [YA]; age, 20–49 years) and after (older adults [OA]; age, 50–88 years) the vertex (r = 0.130 for YA and r =  − 0.388 for OA; test for difference between correlation coefficients, *P* < 0.001). This difference remained significant after adjusting for sex and average steps (r = 0.091 for YA and r =  − 0.323 for OA; test for difference between correlation coefficients, *P* < 0.001). Therefore, we divided the participants into YA and OA groups.

Table 1 shows participant characteristics of the YA (n = 255, 62.4% women) and OA (n = 444, 62.2% women) groups. Mean age was 38.8 ± 6.7 years for YA and 63.6 ± 8.0 years for OA. OA had slower DGS (*P* < 0.001, Cohen’s d = 0.30), and higher VFA (*P* < 0.001, Cohen’s d = 0.28) and BMI (*P* < 0.001, Cohen’s d = 0.17) than YA. The proportion of abdominal obesity was significantly higher in OA than in YA (*P* = 0.048), whereas the proportion of general obesity was not significantly different. No significant differences were found in the accelerometer wear time. Smoking was less prevalent in OA (*P* = 0.005, Cohen’s d = 0.15) and this group had a higher total energy intake than YA (*P* < 0.001, Cohen’s d = 0.24); alcohol intake was comparable. The proportions of hypertension, diabetes, and dyslipidaemia were higher in OA than in YA (all *P* < 0.001).

**Table 1:** Participant characteristics stratified based on age group.

	Younger adults (n = 255)	Older adults (n = 444)	*P* value	Cohen’s d
Age (years)	38.8 ± 6.7	63.6 ± 8.0	< 0.001	3.36
Sex (% women)	62.4	62.2	0.960	
Height (cm)	164.5 ± 7.7	159.0 ± 8.2	< 0.001	0.69
Body weight (kg)	61.1 ± 13.5	58.4 ± 9.7	0.106	0.23
Body mass index (kg/m^2^)	22.4 ± 3.9	23.0 ± 3.0	< 0.001	0.17
Visceral fat area (cm^2^)	73.6 ± 48.1	85.8 ± 39.3	< 0.001	0.28
General obesity (%)	20.0	24.3	0.189	
Abdominal obesity (%)	24.7	31.8	0.048	
Hypertension (%)	19.2	54.7	< 0.001	
Diabetes (%)	2.7	10.4	< 0.001	
Dyslipidemia (%)	24.7	50.0	< 0.001	
Daily gait speed (m/s)	1.27 ± 0.18	1.22 ± 0.20	< 0.001	0.30
Average steps (/day)	6573 ± 2732	6669 ± 2809	0.599	0.03
HW-100 wear time (hour/day)	15.7 ± 2.0	15.6 ± 1.8	0.626	0.04
Smoking habit (cigarettes/day)	3 ± 6	2 ± 6	0.005	0.15
Total energy intake (kcal/day)	1762 ± 536	1890 ± 557	< 0.001	0.24
Alcohol intake (g/day)	12.5 ± 22.0	13.2 ± 23.1	0.732	0.03

Values are the mean ± SD or percentages. Mann–Whitney U tests were used for continuous variables and Chi-square tests were used for categorical variables.

The effect of age on the association between DGS and VFA or BMI was analysed. There was a significant difference in VFA between the two groups (r = 0.099 for YA and r =  − 0.080 for OA; test for difference between correlation coefficients, *P* = 0.023; Fig. 1b). Similar results were obtained for BMI (r = 0.067 for YA and r =  − 0.124 for OA; test for difference between correlation coefficients, *P* = 0.015; Fig. 1c).

To evaluate the association between DGS and abdominal and general obesity, we divided the participants into quartiles according to their DGS, specifically, DGS < 1.11, 1.11 ≤ DGS < 1.23, 1.23 ≤ DGS < 1.37, and DGS ≥ 1.37. The distribution of YA and OA in the overall DGS quartiles is shown in Table 2. In OA, the proportion decreased from quartile 1 to 4, whereas it increased in YA. The proportion of OA in quartile 1 was significantly higher than that of YA (*P* < 0.001), and the proportion of OA in quartile 4 was significantly lower than that of YA (*P* = 0.034).

**Table 2:** The distribution of younger adults and older adults in the overall daily gait speed quartiles.

	Cut-points	Younger adults (n = 255)	Older adults (n = 444)	*P*-value
	m/s	% (n)	% (n)	
Quartile 1	< 1.11	17.3 (44)	29.5 (131)	< 0.001
Quartile 2	1.11–1.23	25.9 (66)	24.5 (109)	0.764
Quartile 3	1.23–1.37	27.1 (69)	23.6 (105)	0.361
Quartile 4	≥ 1.37	29.8 (76)	22.3 (99)	0.034
*P* for trend		< 0.001	< 0.001	

Equality of proportions was analysed. Each age in the four groups was compared using Cochran-Armitage trend tests.

The aOR for the associations between DGS and abdominal or general obesity are presented in Table 3. In the OA, the highest quartile of DGS significantly decreased with the aOR of abdominal obesity compared to the lowest quartile in all models (Model 4: aOR = 0.40 [95%CI 0.18, 0.88], *P* = 0.022). There was no significant association with abdominal obesity in quartile 2 (Model 4: aOR = 1.02 [95% CI 0.51, 2.01], *P* = 0.964) or quartile 3 (Model 4: aOR = 0.86 [95% CI 0.43, 1.72] *P* = 0.669) compared with that of the lowest quartile in all models. DGS was significantly and negatively associated with abdominal obesity when analysed as a continuous variable per 0.1 m/s (Model 4: aOR = 0.86 [95% CI 0.74, 0.99] *P* = 0.035). General obesity showed similar results in OA; only the highest quartile had a significantly decreased aOR compared with that of the lowest quartile in all models (Model 4: aOR = 0.28 [95% CI 0.13, 0.61], *P* = 0.001), DGS as a continuous variable was also significantly associated with general obesity (Model 4: aOR = 0.82 [95% CI 0.72, 0.94] *P* = 0.005). However, these significant associations between DGS and both abdominal and general obesity were not observed in YA.

**Table 3:** Adjusted odds ratio for daily gait speed quartile with abdominal obesity or general obesity.

	Younger adults					Older adults				
	Quartile 1	Quartile 2	Quartile 3	Quartile 4	Continuous (per 0.1 m/s)	Quartile 1	Quartile 2	Quartile 3	Quartile 4	Continuous (per 0.1 m/s)
**Abdominal obesity**										
Model 1	1.00 (ref.)	0.97 (0.33, 2.91)	1.45 (0.52, 4.01)	1.04 (0.38, 2.88)	0.98 (0.82, 1.16)	1.00 (ref.)	1.03 (0.54, 1.97)	0.77 (0.40, 1.51)	0.42 (0.20, 0.86)*	0.86 (0.76, 0.98)*
Model 2	1.00 (ref.)	0.97 (0.32, 2.91)	1.42 (0.50, 4.02)	1.03 (0.37, 2.87)	0.97 (0.81, 1.16)	1.00 (ref.)	1.04 (0.54, 2.00)	0.77 (0.39, 1.51)	0.42 (0.20, 086)*	0.86 (0.75, 0.98)*
Model 3	1.00 (ref.)	0.88 (0.29, 2.66)	1.41 (0.50, 3.98)	1.05 (0.38, 2.93)	0.98 (0.82, 1.17)	1.00 (ref.)	1.04 (0.54, 2.00)	0.78 (0.40, 1.54)	0.44 (0.21, 0.93)*	0.87 (0.76, 0.999)*
Model 4	1.00 (ref.)	0.79 (0.23 2.71)	1.39 (0.44, 4.38)	1.11 (0.36, 3.47)	0.98 (0.80, 1.20)	1.00 (ref.)	1.02 (0.51, 2.01)	0.86 (0.43, 1.72)	0.40 (0.18, 0.88)*	0.86 (0.74, 0.99)*
**General obesity**										
Model 1	1.00 (ref.)	1.05 (0.41, 2.66)	0.72 (0.28, 1.87)	0.44 (0.16, 1.17)	0.83 (0.69, 0.99)*	1.00 (ref.)	0.86 (0.48, 1.55)	0.58 (0.31, 1.10)	0.31 (0.15, 0.65)**	0.84 (0.74, 0.95)**
Model 2	1.00 (ref.)	1.05 (0.41, 2.69)	0.83 (0.31, 2.17)	0.45 (0.17, 1.23)	0.84 (0.71, 1.01)	1.00 (ref.)	0.83 (0.46, 1.51)	0.55 (0.29, 1.03)	0.30 (0.14, 0.63)**	0.83 (0.73, 0.94)**
Model 3	1.00 (ref.)	1.00 (0.39, 2.56)	0.83 (0.32, 2.18)	0.47 (0.17, 1.27)	0.85 (0.71, 1.02)	1.00 (ref.)	0.83 (0.46, 1.51)	0.55 (0.29, 1.04)	0.30 (0.14, 0.65)**	0.83 (0.73, 0.95)**
Model 4	1.00 (ref.)	0.91 (0.33, 2.52)	0.72 (0.25, 2.06)	0.43 (0.14, 1.25)	0.83 (0.68, 1.01)	1.00 (ref.)	0.82 (0.44, 1.51)	0.60 (0.31, 1.15)	0.28 (0.13, 0.61)**	0.82 (0.72, 0.94)**

Values are adjusted odds ratios (95% CI). Multiple logistic regression was performed. Model 1 was adjusted for age and sex. Model 2 was adjusted for model 1 plus smoking habits, energy intake, and alcohol intake. Model 3 was adjusted for model 2 plus average steps/day. Model 4 was adjusted for model 3 plus hypertension, diabetes, and dyslipidaemia. *P < 0.05, **P < 0.01.

## Discussion

This is the first study to investigate the effect of age on the association between DGS and abdominal obesity, as well as general obesity. We found that the tendency of DGS was changed before and after the vertex age (50 years). Therefore, we divided the participants into two groups and found that the associations between DGS and VFA or BMI were significantly different between YA and OA (Fig. 1b,c). One possible explanation for this difference is that the energy expenditure of daily walking differs with age. A previous meta-analysis indicated that elderly participants (mean age ≥ 59 years) expend more energy than younger participants (mean age 18–41 years) when walking at comparable speeds^6^. Ortega et al.^39^ demonstrated that elderly participants (mean age 76 years) expended 34% more metabolic energy while walking than younger participants (mean age 25 years). Therefore, age differences might affect the associations between DGS and VFA or BMI.

In the present study, higher DGS was associated with a significantly lower aOR of abdominal and general obesity in OA. Several studies have demonstrated that the energy cost of walking depends on speed^11–13^. Ortega et al. demonstrated that this dependence was stronger in elderly subjects^39^. Therefore, the present study demonstrated that faster walking might contribute to the prevention of abdominal and general obesity in OA, but not in YA. Although we adjusted for several potential variables that have been reported to be associated with obesity, other age-related variables such as gut microbiota may mediate the link between DGS and obesity through energy metabolism^40,41^. Another possible hypothesis is that a gradual loss of muscle fibres and motor units, which begins at approximately 50 years of age^42^, affects energy expenditure thereby modifying the relationship between DGS and obesity in OA.

Several studies have reported an association between gait speed and abdominal or general obesity; however, the results were inconsistent. Ko et al.^18^ reported significant associations between general obesity and decreased gait speed in subjects aged 50–84 years. Similar results were also reported by other studies on elderly subjects^14–16^. However, Moreira et al.^19^ found no significant association between gait speed and abdominal obesity in middle-aged subjects aged 40–65 years. For DGS, Schimpl et al.^20^ reported no significant association between DGS and general obesity in their study population, which comprised individuals within a broad age range, of 17–65 years. One of the reasons for these inconsistent results might be that these studies did not stratify the subjects by age. In the present study, we divided our participants into two groups (YA, 20–49 years and OA, 50–88 years), and found that the association between age and DGS was significantly different between YA and OA. We also observed that DGS clearly decreased with age in OA (Fig. 1a). These data indicated that there might be an important change between the middle and older ages (e.g. a loss of muscle fibres^42^), which caused the decline in DGS, thereby affecting the association between DGS and abdominal or general obesity.

Abdominal obesity increases with age^43^, whereas gait speed decreases^20,21^. In our study participants, OA had significantly higher proportions of abdominal obesity than YA. The DGS of OA was significantly lower than that of YA, which led to a higher proportion of OA in quartile 1 (DGS < 1.11 m/s) and a lower proportion of OA in quartile 4 (DGS ≥ 1.37 m/s) compared to YA. As the world population grows older^44^, the population with lower DGS suffering from abdominal obesity might increase in the future. Therefore, DGS is expected to be one of the useful indicators for preventing abdominal obesity in people over 50 years of age. However, further studies are warranted to validate these findings.

Several studies describing the association between gait speed and health status were well summarised by Middleton et al.^45^, and gait speed was regarded as a functional vital sign. Sun et al.^46^ demonstrated that women whose walking pace was brisk or very brisk (≥ 1.34 m/s) had 2.68-fold increased odds of successful aging compared to women with an easy walking pace. There are several reports for setting cut-off points, especially for elderly people. For example, there was a lower risk of events and better survival at a gait speed > 1 m/s and even better prospects for extremely fit individuals (gait speed > 1.3 m/s)^45,47^. However, it is noteworthy that these cut-off points are assessed by gait speed in laboratory settings (in-laboratory gait speed). Takayanagi et al.^21^ demonstrated a weak association (r = 0.333, *p* < 0.001) between DGS and in-laboratory gait speed; therefore, we cannot simply compare these cut-off points with DGS. However, although a few studies reported cut-off points assessed using DGS, the present study demonstrated that DGS > 1.37 m/s might lead to decreased abdominal and general obesity in elderly people.

The strengths of this study include the relatively large number of samples with a wide range of ages and the objectively measured DGS assessed by a tri-axial accelerometer. Despite these strengths, our study has several limitations. First, the cross-sectional design of our study could not establish a causal relationship between DGS and obesity status. A longitudinal study would be necessary to determine causality and the associated mechanisms. Second, although similar participation rates have been reported in other studies that have used accelerometers^48^, the loss of participants due to insufficient accelerometer data could have resulted in a selection bias in this study. Finally, as this study was confined to participants from a particular country, region, and race, reproducibility should be confirmed by the inclusion of participants from different regions and/or races.

## Conclusions

In conclusion, the association between DGS and abdominal and general obesity differed significantly by age. In OA, DGS was significantly and negatively associated with abdominal obesity and general obesity, whereas no significant associations were found in YA. These data could aid in raising awareness about the self-management of obesity via DGS monitoring, especially in case of OA. The effect of age on the relationship between DGS and obesity warrants further investigation.
